# Mechanical mechanics-reclaiming a new battlefield for chronic liver disease

**DOI:** 10.1016/j.jare.2025.05.028

**Published:** 2025-05-14

**Authors:** Yiheng Zhang, Tianle Ma, XingXing Lu, Haibing Hua, Li Wu, Zhipeng Chen

**Affiliations:** a Jiangsu Key Laboratory for Pharmacology and Safety Evaluation of Chinese Materia Medica, School of Pharmacy, Nanjing University of Chinese Medicine, Nanjing 210023, China; b Department of Gastroenterology, Jiangyin Hospital of Chinese Medicine Affiliated to Nanjing University of Chinese Medicine, Nanjing University of Chinese Medicine, Jiangyin 214400, China; c Engineering Center of State Ministry of Education for Standardization of Chinese Medicine Processing, School of Pharmacy, Nanjing University of Chinese Medicine, Nanjing 210023, China

**Keywords:** Chronic liver disease, Mechanical mechanics, Liver sinusoid, Hepatic stellate cell, Myofibroblast-like transition

## Abstract

**Background:**

In the 21st century, significant breakthroughs have been made in the research of chronic liver disease. New biochemical markers of pathogenicity and corresponding drugs continue to emerge. However, current treatment strategies remain unsatisfactory due to complex pathological changes in the liver, including vascular dysfunction, myofibroblast-like transition, and local tissue necrosis in liver sinusoids. These challenges have created an urgent need for innovative, synergistic treatments. Mechanical mechanics is a growing field, with increasing evidence suggesting that mechanical signals play a role similar to that of biochemical markers. These signals influence response speed, conduction intensity, and functional diversity in regulating cell activities.

**Aim of review:**

This review summarizes the three main mechanical characteristics involved in the progression of “liver fibrosis-cirrhosis-hepatocellular carcinoma” and provides an in-depth interpretation of several mechanically-related targets. Finally, current and cutting-edge therapeutic strategies are proposed from a cellular perspective. Despite the many challenges that remain, this review is both relevant and significant.

## Highlights

• Mechanical mechanics plays an irreplaceable role in chronic liver disease (CLD) due to traditional biochemical factors.
• ECM cross-linking and liver tissue sclerosis, shear stress, and cellular phenotypic changes are the three major culprits for CLD.
• A series of classical and newly discovered mechanical mechanics targets form a complex network of stress and biochemical signals acting together.
• Some intercellular multi-mechanism therapeutic strategies are proposed, which are expected to indicate the future of CLD.

## Graphical abstract

**Figure:**
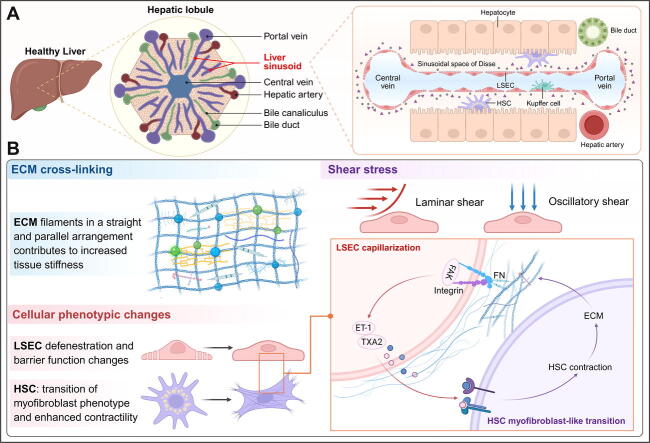

## Introduction

According to data from the International Agency for Research on Cancer (IARC), more than 840,000 new cases of hepatocellular carcinoma (HCC) are reported globally each year [1]. HCC ranks sixth among malignant tumors and fourth in terms of mortality. Clinically, approximately 80 % of HCC patients have a background of chronic liver disease (CLD), such as liver fibrosis and cirrhosis [2]. Unfortunately, there are over 400 million CLD patients in China alone, underscoring the need for early intervention and prevention of HCC.

Despite significant advances in the understanding of CLD mechanisms and treatment strategies across multiple areas (Table 1), clinical outcomes often remain unsatisfactory. Western medicine alone, or the combination of Western and traditional Chinese medicine (TCM), frequently fails to achieve optimal results.

**Table 1:** Popular mechanisms and mainstream treatment strategies of CLD.

Main mechanisms	Strategies or drugs	References
Oxidative stress, inflammation	Resveratrol, Quercetin, Hydroxysafflor yellow A etc., enhance antioxidant enzyme activity or inhibit inflammatory signaling pathways.	[[3], [4], [5]]
Intracellular overloaded iron or copper etc.	Deferoxamine mesylate salt, liproxstatin-1, etc., celate iron or copper, or inhibit ferroptosis and cuproptosis in hepatocytes. Oroxylin A, etc., exacerbate frroptosis in HSCs.	[[6], [7], [8], [9], [10]]
Disorders of glycometabolism and lipometabolism	Lanifibranor, Insulin, etc., interference with PPARs or PI3K/AKT.	[[11], [12], [13]]
Vascular networks disorder	Rhein, Naringenin, Miransertib, etc., suppress pathological angiogenesis or alleviate vascular inflammation and leakage.	[[14], [15], [16], [17], [18], [19]]
Abnormal immune microenvironment or TME	Bacteroides acidifaciens, intestinal flora, Pembrolizumab, etc., aid detoxification of hepatocyte or as anti-PD-1/PD-L1 immunotherapy.	[[20], [21], [22]]
Liver injury induced by drugs, alcohol, etc.	Colchicine, Glycyrrhizin, Silymarin, etc., protect hepatocyte and reduce transaminases	[23,24]

Recently, the intersection of biomedicine and physics has brought increased attention to the mechanical mechanics underlying CLD and HCC [18,25,26]. From the perspective of the physical characteristics of the liver, the liver plate and sinusoids are surrounded by extracellular matrix (ECM), and tissue stiffness progressively increases with liver fibrosis [26]. This stiffness is accompanied by changes in cellular phenotypes and functions, indicating multiple mechanistic control points [[27], [28], [29]]. It has been confirmed that mechanical and chemical signaling interact to synergistically drive disease progression [27,30]. Recent clinical studies [[31], [32], [33]] have confirmed that liver stiffness is significantly positively correlated with the risk of liver-related events (such as decompensation of liver cirrhosis, HCC, liver disease death). It is a key predictor of clinical outcomes in non-alcoholic fatty liver disease (NAFLD), viral hepatitis, and autoimmune hepatitis. This review focuses on the patterns and complex mechanisms of liver mechanical signaling in CLD progression. We also highlight key insights into some classic and recently discussed small molecules and signaling pathways. Additionally, we analyze feasible therapeutic strategies for CLD and HCC from a multicellular perspective.

## Mechanical stress drives CLD

**Fig. 1:**
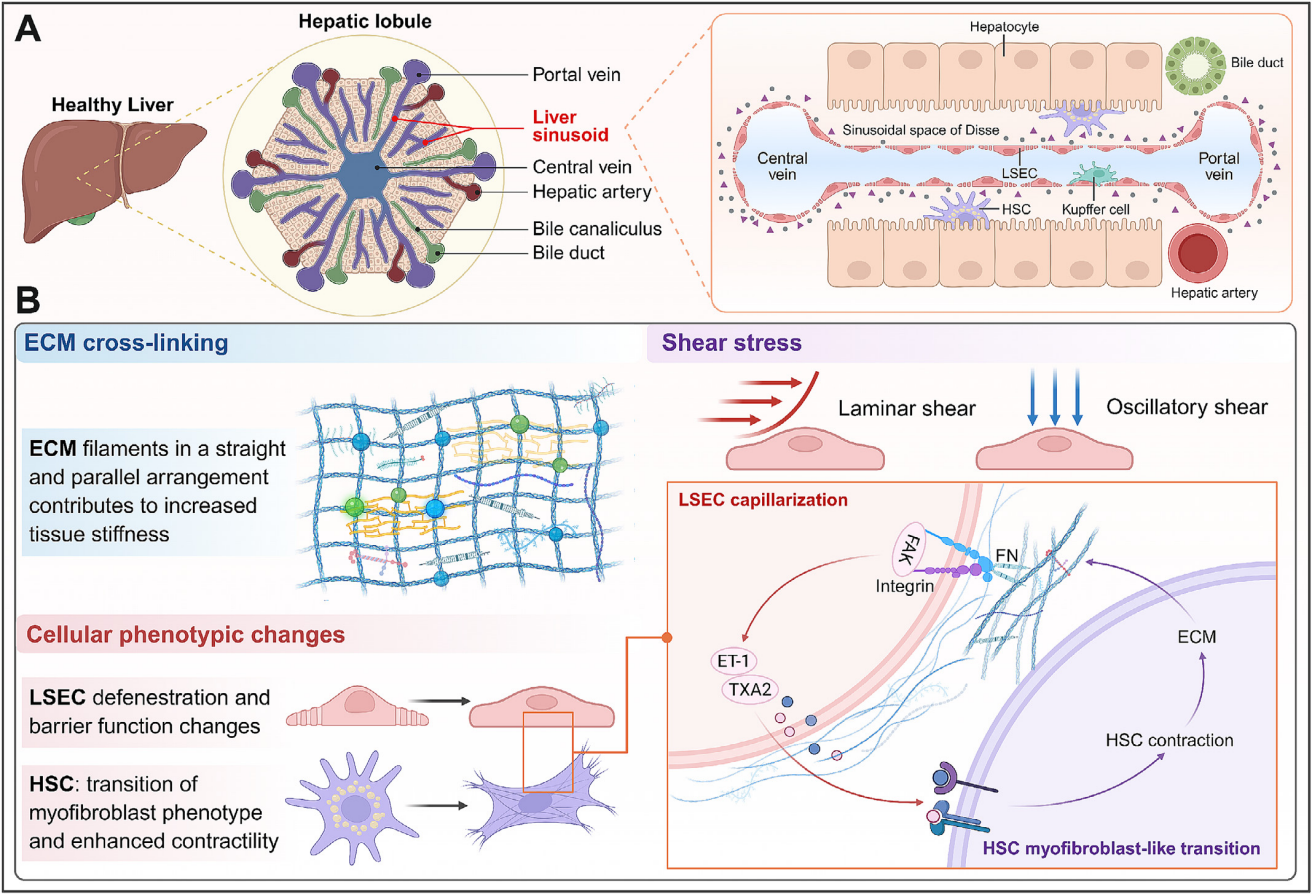
**(Graphical abstract).** Key mechanical cues influencing the mechanical microenvironment of liver sinusoids. (A) Schematic diagram of liver sinusoids in normal mice. (B) ECM cross-linking, shear stress, and cellular phenotypic changes are three critical mechanical cues in the progression of CLD. This figure was created in BioRender. Tianle, M. (2025) https://BioRender.com/re6kihw.

The concept of “biomechanics”, introduced by Feng Yuanzhen in the 1960s [34], has evolved into the field of mechanobiology, which gained significant attention at the World Congress of Biomechanics held in Munich, Germany, in the early 21st century. Mechanobiology refers to the effects of mechanical environments (stimulation) on health, disease, or injury, as well as the mechanisms by which organisms perceive and respond to mechanical signals. It also explores the mechanical and biological processes within organisms [35]. Currently, the mechanophysical properties of tumors are summarized as elevated solid stress, increased interstitial fluid pressure, heightened stiffness, altered material properties, and changes in tissue microarchitecture [36]. By combining the structure of liver sinusoids with the pathological features of CLD, we categorize the mechanical concepts related to these conditions into three main areas: ECM cross-linking and liver tissue sclerosis (HSC is the main contributor), shear stress (LSEC suffers the most direct stress), and cellular phenotypic changes (HSC and LSEC are the main effector cells) (Fig. 1). These three areas operate in parallel and crosstalk during CLD progression. Recently, we performed single-cell sequencing of liver cirrhosis mice, revealing that liver sinusoidal endothelial cell (LSEC) and hepatic stellate cell (HSC) are upregulated in both number and intensity of interactions. HSC-derived ECM components, such as collagen, fibronectin (FN), laminin, and vitronectin, interact with LSEC through the “ECM-receptor” mode. In return, LSEC overexpression of ET-1, TXA2, and other components affects HSC, resulting in the loss of both HSC myofibroblast-like transition and LSEC capillarization. Although more details need to be confirmed, these results shed light on the response strategies and intrinsic connections of molecular signaling networks in tissues and cells under mechanical stress during CLD progression. This also aids in understanding the mechanical mechanisms that drive liver function transitions from “compensation” to “decompensation”.

### ECM cross-linking and biomechanical properties of liver tissue

Abnormal ECM deposition is the earliest and most significant factor that increases liver solid stress and tissue stiffness, alters the mechanical microenvironment of liver sinusoids, elevates interstitial fluid pressure, and drives the ongoing progression of CLD. The reason why increased liver stiffness threatens patient survival [37] is that ECM not only alters tissue stiffness but also creates an ischemic-hypoxic environment that severely impairs normal cellular metabolism. Moreover, it restricts drug delivery and even protects cancer cells from treatment.

Physiologically, ECM follows a tightly regulated mechanism: newly secreted ECM undergoes cross-linking via lysyl oxidases (LOX) and transglutaminases (TGM) [38,39], providing an environment conducive to cell survival and activity. This process influences cell shape, metabolism, function, migration, proliferation, and differentiation through signal transduction systems. Meanwhile, HSC express matrix metalloproteinases (MMPs) to promote ECM degradation, maintaining a dynamic equilibrium. As a result, normal human liver stiffness ranges from 2.0 to 7.3 kPa [25]. However, when liver injury occurs due to various causes, the dynamic balance between ECM synthesis and degradation is disrupted, and ECM deposition continues unchecked [40]. The cross-linking of deposited ECM progressively increases tissue stiffness, which is sensitively detected by HSCs, keeping them in a long-term activated state until apoptosis. When liver stiffness reaches 12.5 kPa, it signals that CLD has advanced to its terminal stage-cirrhosis-and indicates an increased risk of cancerous transformation [25]. Thus, ECM is considered a major driver of fibrosis and sclerosis [39].

**Fig. 2:**
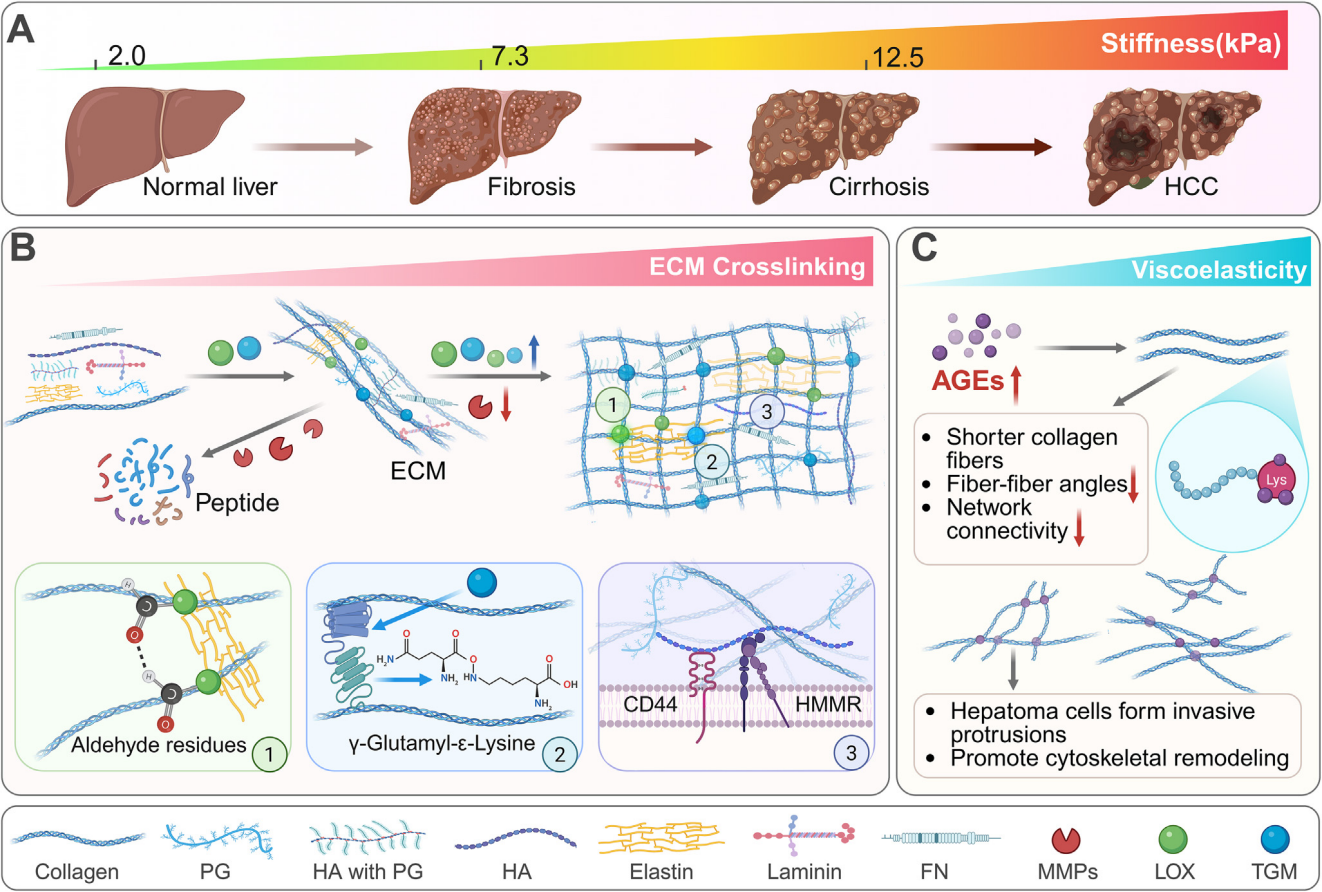
ECM cross-linking and subsequent deposition correlate positively with increased liver tissue stiffness. (A) Liver stiffness is a critical clinical measure of liver physiology, closely associated with poor prognosis. Thresholds of 7.3 kPa and 12.5 kPa are clinically recognized for diagnosing liver cirrhosis and HCC, respectively. (B) ECM is a dynamic entity involving complex components and mechanisms, ranging from secretion to cross-linking, solidification, and deposition. Key contributors to ECM cross-linking include the highly reactive aldehyde residues of LOX and TGM and γ-Glutamyl-ε-Lysine cross-links, facilitating the binding of collagen fibers to elastin and to each other. HA physically “bundles” cells and ECM through tethered interactions. FN also bridges collagen and integrins to complicate ECM. Liver stiffness accumulates from these processes, a common phenomenon in fibrotic organs. (C) Viscoelasticity, a physical property distinct from liver stiffness, is also linked to poor prognosis in HCC patients. Increased viscoelasticity is primarily observed in CLD patients with T2DM. Its pathological product, AGEs, interacts with lysine residues, disrupting the structural integrity of the collagen network and leading to shorter collagen fibers with decreased inter-fiber angles. This figure was created in BioRender. Tianle, M. (2025) https://BioRender.com/f61l655.

In recent years, researchers have increasingly focused on the effects of ECM cross-linking on liver tissue hardness, mechanical-biochemical coupling, and other biomechanical factors in liver tissue. The ECM is a 3D macromolecular cross-linking network primarily composed of collagen, FN, elastin, lamin, proteoglycans, glycoproteins, and glycosaminoglycans [41]. The mechanical properties of ECM are determined by the composition and structure of this network. During CLD progression, ECM composition is reorganized, with a significant increase in collagen types I and III, accompanied by enhanced cross-linking. Additionally, cross-linking of ECM filaments in a straight and parallel arrangement contributes to increased tissue stiffness, primarily driven by LOX and TGM. LOX is a copper-dependent enzyme that initiates collagen and elastin cross-linking through highly reactive aldehyde residues, stabilizing ECM deposition and enhancing tissue stiffness and integrity [42]. TGM promotes the formation of γ-Glutamyl-ε-Lysine cross-linking bonds between adjacent collagen fibers, increasing stability, irreversibility, and resistance to plasticity [43]. HA is a component of ECM that binds to cells via interactions with membrane receptors CD44 and the HA-mediated motility receptor (HMMR) [44], enhancing cell adhesion and contractility. TGF-β is another potent pro-fibrotic factor present within ECM. It not only directly participates in ECM cross-linking but can also be released from ECM in response to mechanical signals generated by cross-linking [45]. Clinical samples (https://timer.cistrome.org/) have shown that the genes for LOX, TGM, and HA are overexpressed in HCC, contributing to sustained liver stiffness. More importantly, increased ECM stiffness stimulates mechanical-biochemical coupling signaling pathways, such as integrin-FAK-Rho GTPase, RhoA/ROCK/Myosin/YAP, TGF-β, and Wnt/β-catenin. These pathways significantly contribute to cell proliferation, vascular remodeling, cancer cell migration, and immune escape [41] (Fig. 2).

Recently, a new mechanical factor, viscoelasticity, has been found to promote HCC independently of stiffness. Clinically, liver fibrosis patients with a background of type 2 diabetes mellitus (T2DM) are more likely to develop HCC before progressing to cirrhosis. This is due to the interaction of advanced glycation end-products (AGEs) with lysine residues of collagen telopeptides, which interferes with fiber elongation and the formation of structurally cohesive collagen networks. As a result, shorter collagen fibers in ECM reduce fiber–fiber angles and network connectivity. ECM with high viscoelasticity is more capable of inducing changes in hepatoma cell shape, promoting the formation of invasive protrusions, aiding cell escape, and enhancing invasiveness. Among the ECM components, FN stands out for its special flexibility and tensile properties, participating in the dynamic adjustment of viscoelasticity [46]. With its unique “V” conformation, FN bridges ECM components (such as collagen) and integrins on the cell membrane. The latest research [47] confirms that conformational changes in FN fibers can directly drive the activation of integrin receptors, promoting integrin-responsive cytoskeletal remodeling, including F-actin polymerization, MLC phosphorylation, and actin contractility. This, in turn, synergizes with stiffness to activate mechanical-biochemical coupling signaling, accelerating disease progression [26].

### The coercive effect of shear on liver sinusoids

During CLD progression, changes in vascular mechanical properties, in addition to increased tissue stiffness, are crucial components of liver mechanical characteristics, particularly in advanced stages of the disease. Liver sinusoids, as specialized capillaries, are constantly exposed to blood flow. The constituent cells benefit from blood-oxygen exchange and are also regulated by shear stress from blood flow. Laminar shear is a force generated by blood flow parallel to the long axis of the vessel, while oscillatory shear refers to the pressure exerted by blood flow on the vessel wall, perpendicular to the long axis, due to gravity. Laminar shear (in pre-decompensated cirrhosis) primarily regulates vascular endothelial homeostasis and immune cell recruitment, whereas oscillatory shear (in pre-decompensated cirrhosis) modulates hepatic parenchymal cell metabolic activity and induces cellular damage under specific conditions [48].

LSEC, as the primary components of the liver microvasculature, directly interact with blood flow. In the early stages of CLD, LSEC phenotypic changes are characterized by defenestration and basement membrane formation (**2.3.1 for details**), progressively impeding the exchange of substances between hepatic parenchymal cells and the bloodstream. This results in energy depletion and oxygen deprivation, triggering powerful compensatory mechanisms in the liver, such as pathological angiogenesis, which enhances blood perfusion and generates unprecedented pressure on the vessel walls (including laminar and oscillatory shear). These changes accelerate further phenotypic alterations in LSEC, forming a vicious cycle of endothelial dysfunction and elevated shear stress [49].

Notably, shear stress plays a significant role in inducing liver sinusoidal thrombosis and promoting portal hypertension (PH) [[50], [51], [52]]. The mechanism involves increased shear activating mechanosensitive Piezo1 on LSEC surfaces, which binds to the Notch1 receptor. This promotes CXCL1 expression via Hes1 and Hey1 stimulation of ADAM10 proteins, attracting neutrophils and leading to the formation of neutrophil extracellular traps (NETs) and the release of immature platelets. Platelet endothelial cell adhesion molecule-1 (PECAM-1), an immunoglobulin superfamily member expressed on LSEC surfaces, is considered a potential target for regulating the mechanical microenvironment of the sinusoids, due to its negative regulatory effect on Piezo1 [53].

**Fig. 3:**
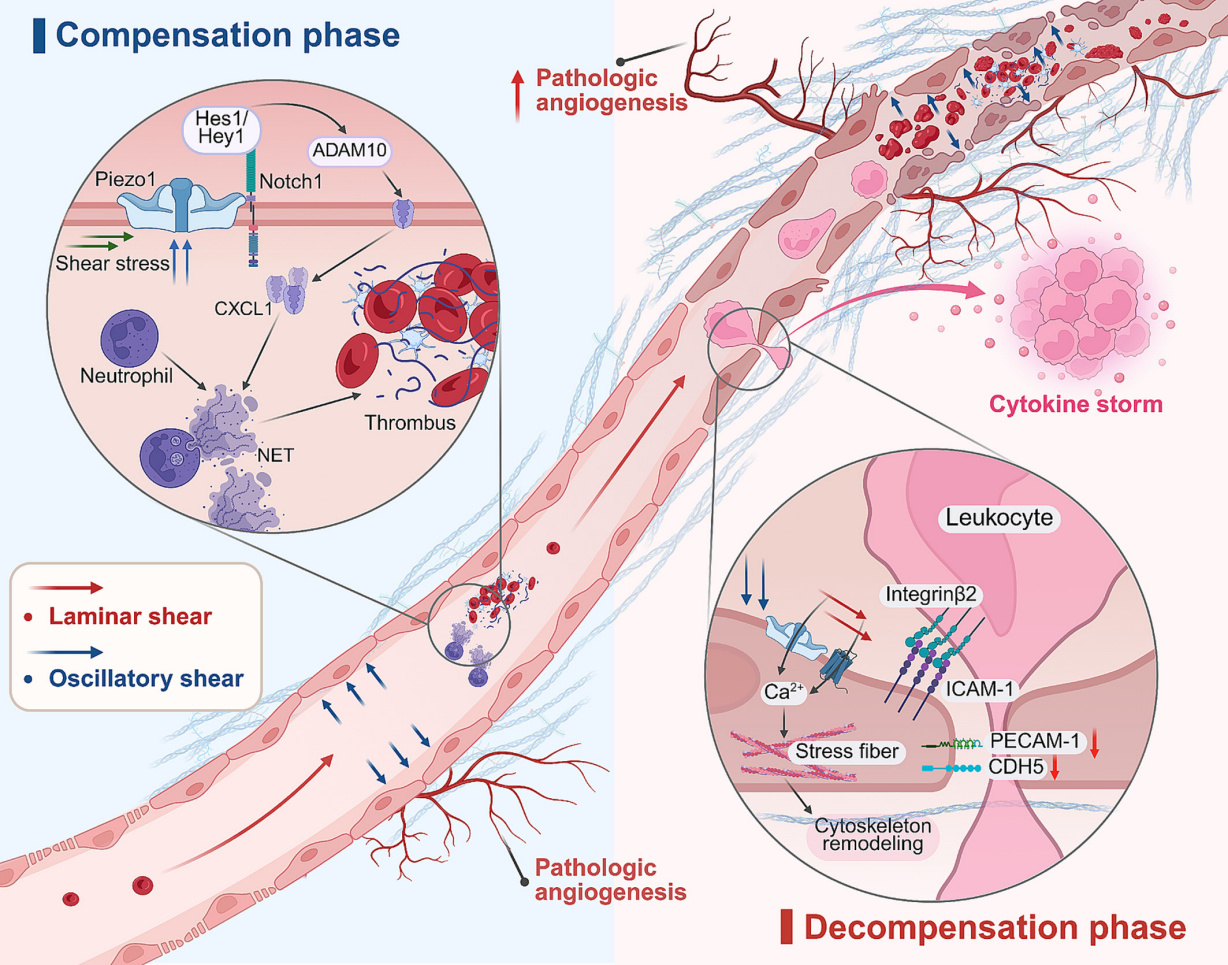
Dynamically changing blood flow shear stress in CLD imposes strain to the liver sinusoidal microenvironment, inducing pathological changes such as defenestration, pathologic vascular proliferation, and inflammatory storms. During the compensation phase (Left), laminar and oscillatory shear persistently impact the vasculature, causing a variety of mechanical secondary injuries including liver sinusoidal thrombosis and NETs. The dual mechanical coercion of shear stress and ECM stiffness induces local ischemia and hypoxia, promoting pathological vascular proliferation. In the decompensation phase (Right), there is a general slowdown in pathological vascular proliferation. Laminar shear ceases to be the predominant stressor; instead, oscillatory shear becomes the primary stressor on the vessel walls, contributing to an ongoing inflammatory storm that further exacerbates endothelial dysfunction, rendering CLD difficult to reverse. This figure was created in BioRender. Tianle, M. (2025) https://BioRender.com/a62g127.

In the decompensation phase of cirrhosis, microvascular proliferation tends to be attenuated (mechanisms remain unclear), which fails to meet the needs for material exchange and oxygenation [54]. Increased intravascular resistance due to stenosis and thrombus leads to stagnant blood flow. This weakens laminar shear but enhances oscillatory shear, which in turn suppresses VE-calmodulin (CDH5) and PECAM-1 expression in LSEC, reducing intercellular adhesion junctions and impairing endothelial barrier function, thus increasing permeability. Additionally, ICAM-1, a ligand for leukocyte integrin β2, regulates leukocyte migration. Vascular embolization and ICAM-1 aggregation synergistically increase endothelial membrane tension, promoting massive leukocyte extravasation, inducing an inflammatory storm, and exacerbating PH and endothelial damage [55]. This positive feedback loop leads to an irreversible increase in local pressure [56], explaining the difficulty in reversing cirrhosis decompensation. Furthermore, several substances on LSEC surfaces, such as Ca^2^^+^ ion channels, G-protein-coupled receptors (GPCRs), and adhesion proteins, all possess mechanosensing capabilities to convert shear stress into intracellular signals involved in vascular endothelial dysfunction [54]. Additionally, increased shear stress in liver sinusoidal vasodilatation promotes LSEC mechanosensitive receptor expression and cytoskeletal remodeling, transmitting extracellular mechanical signals to the cell. This significantly increases the secretion of the pro-cancer substance sphingosine 1-phosphate (S1P), which induces HCC [18]. These findings suggest that shear-mediated mechanical-biochemical coupling signaling plays a critical role in promoting CLD and HCC (Fig. 3).

### Changes in intracellular mechanical properties and cellular phenotypes during CLD progression

#### LSEC defenestration and barrier function changes

LSEC differs from typical vascular endothelial cells due to the absence of septa and the presence of basal fenestrae, which are approximately 50–300 nm in diameter and labyrinthine in structure. These fenestrae connect the lumen of liver sinusoids to the interstitial space of Disse [57]. In CLD, LSEC phenotypic changes are characterized by defenestration and liver sinusoidal capillarization. Defenestration refers to a reduction in the diameter and number of fenestrae in endothelial cells, often accompanied by the formation of a basement membrane. Liver sinusoids themselves are capillaries. When lesions develop on the fenestra and basement membrane, this pathological state is referred to as capillarization [58]. These changes increase shear stress, creating a positive feedback loop between defenestration and enhanced flow shear. Therefore, exploring the biomechanical mechanisms and potential targets of defenestration is crucial to interrupting this vicious cycle [58].

**Fig. 4:**
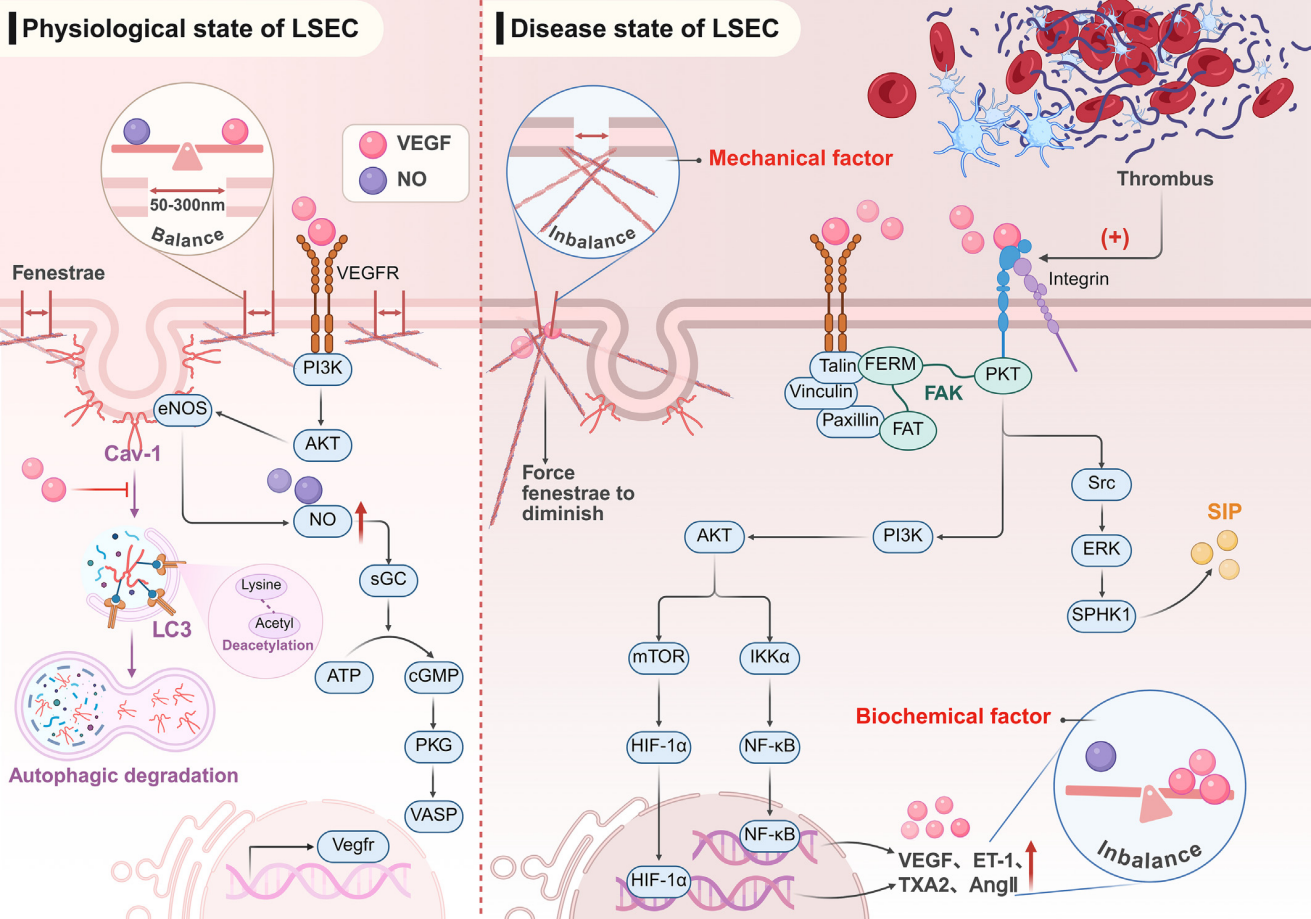
Impact of mechanical stress on LSEC: Loss of defenestration and barrier function changes. (Left) In the normal physiological state, a balanced expression of vasoactive molecules and mechanical homeostasis collaborate to protect the fenestrae and maintain physiological function. (Right) In the disease state, mechanical cues lead to cytoskeletal remodeling and altered expression of vasoactive molecules (notably VEGF), driving the defenestration of LSEC under combined mechanical and biochemical influences. This figure was created in BioRender. Tianle, M. (2025) https://BioRender.com/u67u736.

The liver sinusoids, as the first immune barrier, are the first to activate immune defense during tissue inflammation, albeit at the expense of their structure. For example, ICAM-1, which is upregulated during leukocyte adhesion, not only remodels the cytoskeleton but also induces LSEC surface stiffness, increasing traction stress and promoting junction opening [55]. Caveolin-1 (Cav-1), a versatile membrane lipid raft protein, is essential for maintaining fenestrae in LSEC. Physiological levels of VEGF can preserve fenestrae structure by inhibiting the autophagic degradation of Cav-1 and upregulating NO-dependent pathways (eNOS/NO/sGC/cGMP/PKG/VASP) [59,60]. However, from the early stages of CLD, Cav-1 autophagic degradation and cytoskeletal remodeling occur, continuing throughout the disease process. Regulating the deacetylation of the autophagy marker LC3 can effectively protect fenestrae [61]. Notably, integrins can detect subtle mechanical changes in CLD progression, activating mTOR and IKKα signaling pathways downstream of PI3K/AKT via the FAK domain of PKT. This leads to the dissociation of NF-κB from IκB and the deubiquitination of HIF-1α, increasing the expression of various vasoactive molecules, including VEGF. When VEGF binds to its receptor, it triggers AKT/mTOR and AKT/IKKα signaling cascades through Paxillin and the FAT domain of FAK, forming a positive feedback loop. These factors are critical for inducing defenestration [62]. Paxillin/FAT also contribute to the reduction or disappearance of fenestrae, driven by the synergistic shear forces of VEGF via RhoA/ROCK (with relative expression much lower than in hepatocytes and HSCs). The activated p38 MAPK pathway further promotes the phosphorylation of microtubule-binding proteins and Cofilin, which act as “stabilizing scaffolds” during F-actin polymerization, inducing defenestration following cytoskeletal remodelling [63,64]. Additionally, studies have shown that activation of the integrin β1/FAK/Src signaling pathway can induce Cav-1 Y14 site phosphorylation. On one hand, this inhibits eNOS activity and reduces NO production [65], on the other hand, it regulates MLC phosphorylation, promoting cytoskeleton remodelling [66] (Fig. 4). Notably, integrin/FAK activates the Src/ERK/SPHK1 pathway and promotes S1P expression [18,67], creating a “time bomb” for future carcinogenesis. Piezo1 also plays a crucial role in mechanosensation during this process [68]. Recent research has shown that protein disulfide isomerase (PDI), a protein disulfide bonding enzyme involved in protein folding, reduces disulfide bonds in αIIbβ3 to support platelet aggregation and thrombosis. This either activates integrins (including αvβ3 and α11β1 receptors) or forms a complex directly with β-actin, achieving redox-dependent regulation of cytoskeletal auxiliary actin or upstream signaling molecules (such as Cdc42, Src, or PKC), thus affecting the kinetics of liver sinusoidal fenestrae [69].

#### The pro-fibrotic phenotype transition of HSC

HSC has long been a research focus in CLD, with their transition to a myofibroblast phenotype and increased contractility contributing to disease progression. Although the mechanisms by which chemical mediators such as ET-1, Ang Ⅱ, and Midkine (MK) [27] influence HSC behavior have been widely studied, the role of mechanical-biochemical coupling in triggering HSC contraction and COL1A1 overexpression, as well as its impact on neighboring cells and the liver sinusoidal microenvironment, remains an intriguing topic. In addition to ET-1 and Ang II receptors, mechanosensitive receptors, particularly integrins, have been detected on the HSC membrane (detailed information is provided in Section 3.2). Integrins receive direct mechanical stimulation from intra- and extracellular forces or bind to active molecules, triggering downstream signaling cascades [70]. HSC activation is characterized by ECM deposition, and ECM cross-linking and tissue stiffness provide a “stiff bed” for HSC [71]. The activation of HSC leads to the overexpression of the mechanosensitive protein ROCK, remodeling the cytoskeleton and subjecting HSC to both internal and external mechanical stress. This signal is amplified by Piezo1 and transmitted to integrins through ECM stiffness and cytoskeletal changes [72]. Upon promoting YAP nuclear translocation, the expression of COL1A1 is increased, leading to cell contraction [27].

**Fig. 5:**
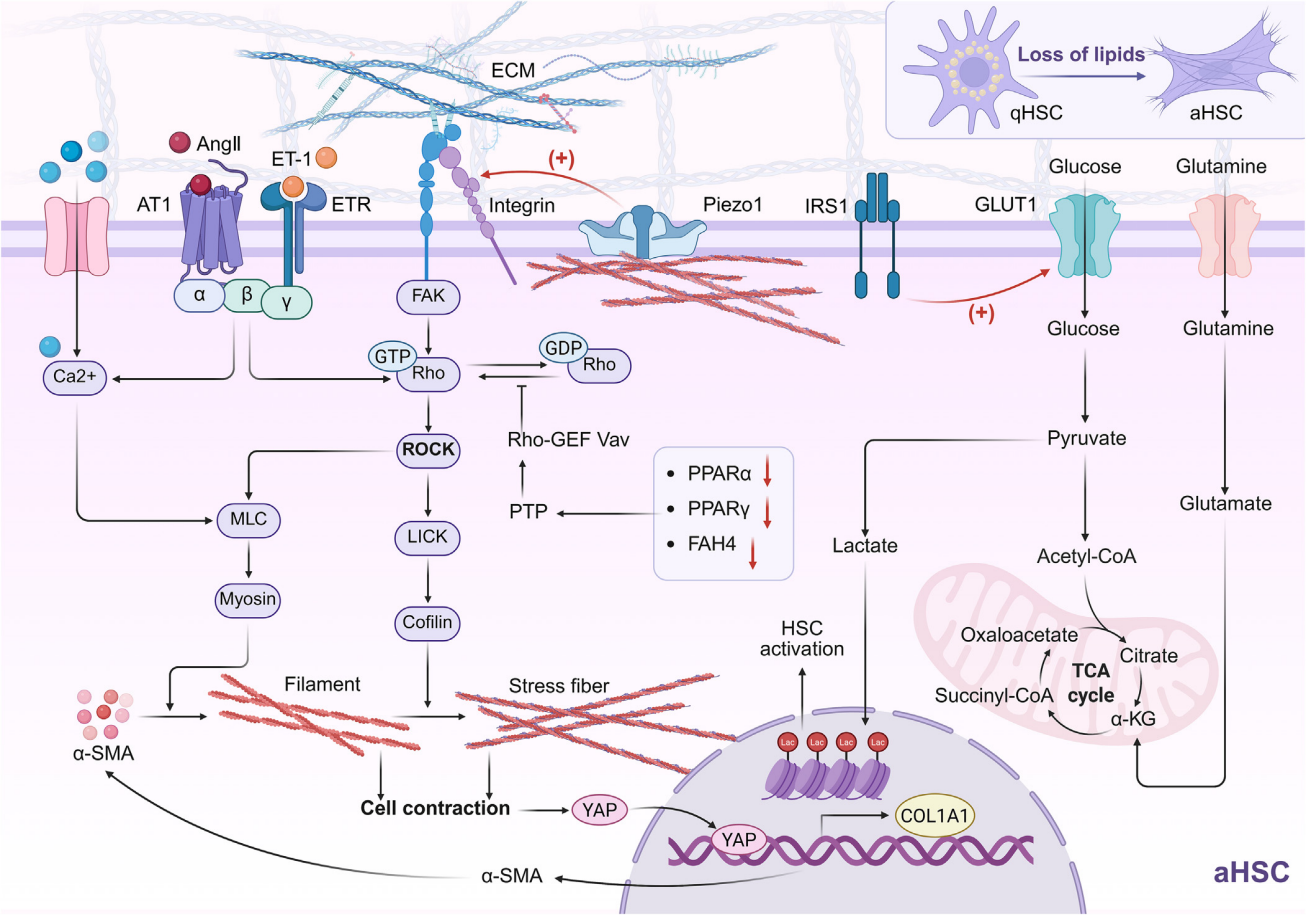
Mechanical cues in the myofibroblast-like transition of HSC. This figure was created in BioRender. Tianle, M. (2025) https://BioRender.com/s64k550.

During the myofibroblast-like transition, metabolic reprogramming (including the loss of lipid droplets, delayed gluconeogenesis, and activation of aerobic glycolysis and glutamine catabolism) affects cell fate by altering intracellular mechanosensitive signalling [73]. Resting-state HSCs resemble adipocytes, and lipid loss is a key marker of HSC activation. Maintaining lipid formation can restore HSC to its resting state. However, metabolic changes occur rapidly within 48 h of transdifferentiation and are sustained in the activated state, manifesting as downregulation of PPARα (involved in lipid oxidation), PPARγ (which promotes adipogenesis), and FABP4 (involved in lipid uptake) [74]. Interestingly, while not yet reported in the liver, it is well established in nerve damage that PPARγ downregulation loses inhibitory control of protein tyrosine phosphatase (PTP). This cytoplasmic PTP inhibits the conversion of GDP-Rho to GTP-Rho by dephosphorylating Rho-GEF Vav [75]. GTP-Rho regulates intracellular mechanical signaling pathways that influence cellular phenotypic transitions. Glycolysis itself is unaffected by cytoskeletal remodeling, but RhoA/ROCK activation secondary to insulin receptor substrate 1 (IRS1) and PI3K p85 increases GLUT1 abundance and glucose uptake, and its metabolite lactate can modify histones to promote HSC activation [76] (Fig. 5). These findings indicate that mechanical signals link glycolipid metabolic alterations to myofibroblast-like transition. Furthermore, glutamine metabolism, active in the liver, is a necessary substrate for myofibroblast-like transition. Its metabolite, α-ketoglutarate (α-KG), can limit the rate of myofibroblast-like transition [77]. Unfortunately, such mechanical-metabolic coupling has been demonstrated in only a few cell types or tissues [78], and further experimental investigation is required for HSC.

#### Polarity transition of hepatocyte

Hepatocytes, the primary component of the liver, have two basal domains facing the liver sinusoid. To ensure proper hepatocyte function, cellular polarity, including both structural and functional components, is tightly regulated. Remarkably, as an innate trait, hepatocytes in embryonic mice acquires polarity by day 14. The structural polarity of hepatocytes includes the morphological integrity of the tight junctions (TJs), the apical plasma membrane, microvilli, and the formation of the BC network. Functional polarity depends on the operation of the tubular ATP-binding cassette (ABC) transporter proteins [79,80].

**Fig. 6:**
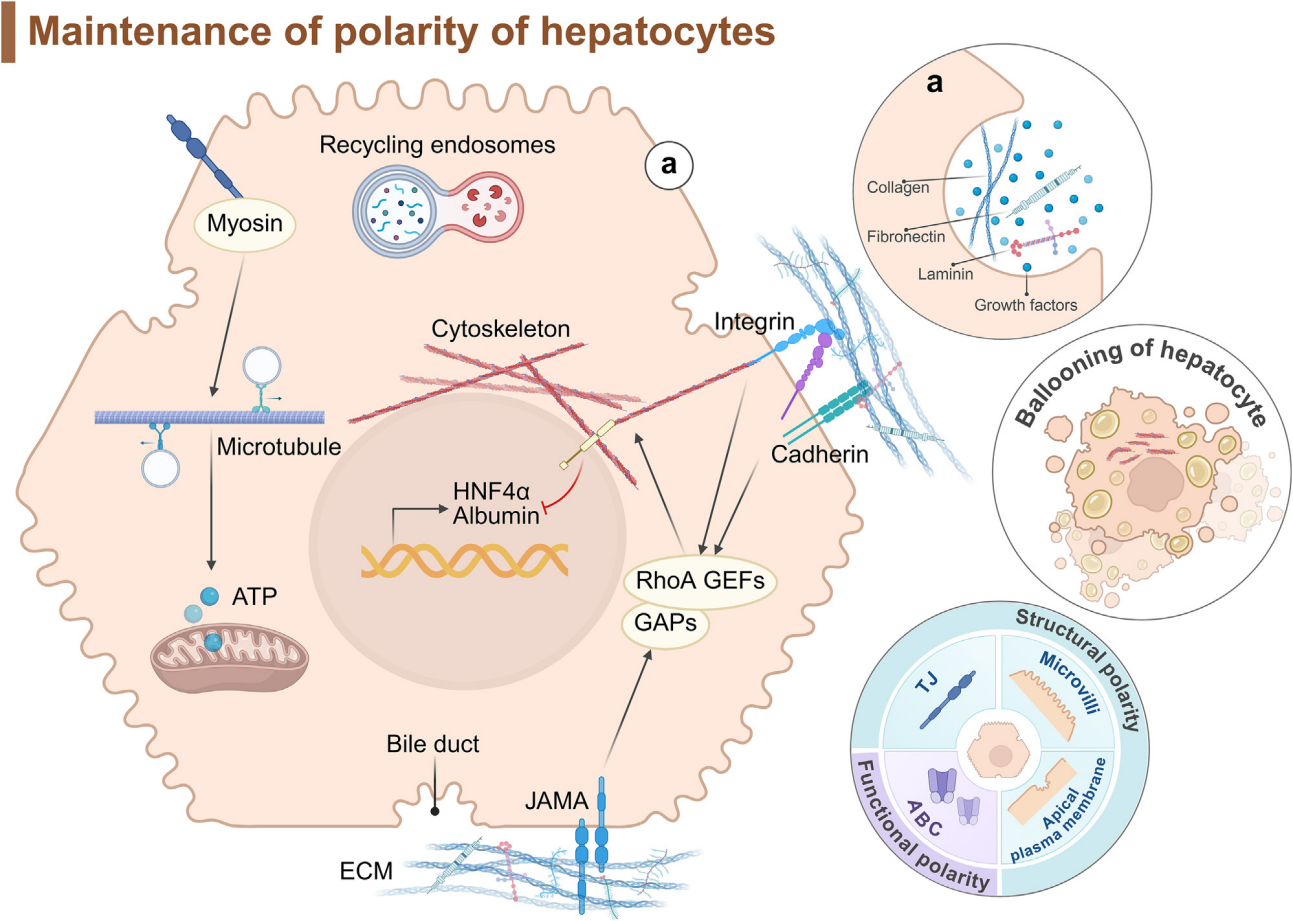
Polarity transition in hepatocyte. (Left) Maintenance of hepatocyte polarity depends on TJ, integrity of the apical plasma membrane, microvilli, and the presence of ABC transporters. (Right) Mechanical cues adversely affect hepatocyte and disrupt the components essential for maintaining polarity. This figure was Created in BioRender. Tianle, M. (2025) https://BioRender.com/q64w416.

Hepatocyte polarization results from the coordination of several key evolutionarily conserved elements. These components include the ECM, adhesion and TJs, and intracellular protein transport mechanisms (including recycling endosomes, cytoskeleton, and ATP) [79]. Collagen, laminin, fibronectin, and growth factors in the ECM are captured by hepatocyte, which are essential for maintaining the polar phenotype [81]. However, cross-linked ECM can decrease or even disrupt this “supply,” and hepatocytes are squeezed by the ECM, leading to changes in cell morphology and nuclear deformation under the action of the cytoskeleton. These changes trigger hepatocyte physiological dysfunction, such as the inhibition of hepatocyte nuclear factor 4α (HNF4α) and albumin expression [82], and a decline in glycogen storage capacity [83]. These alterations are mediated by ECM-driven mechanical signals to RhoA/ROCK, inducing phosphorylation of FAK Tyr397 [83]. Interestingly, lipid metabolism, another metabolic link similar to hepatic glycogen metabolism disorder, is also a common factor disturbing hepatocyte. In fact, NAFLD has become one of the biggest threats to CLD [84]. Hepatocytes are undoubtedly the biggest victims of NAFLD. NAFLD begins with the accumulation of lipid droplets in hepatocyte. The ballooning of hepatocyte caused by the excessive filling of lipid-rich lipid droplets can be transformed into lipotoxicity and destroy the normal structural cytoskeleton. LSEC dysfunction, vascular disorder, ECM deposition, etc. may be involved as the disease progresses [85]. However, the spatiotemporal relationship between steatosis and cytoskeleton remodeling needs further disclosure, which may be a “chicken-or-egg” question (Fig. 6).

#### Mechanical cues of immunocytes

Different subsets of immunocytes regulate fibrosis progression and degeneration [86]. While the relationship between mechanical stress and immunity in the liver remains to be fully explored, it is well-documented. Of particular interest is the ability of various innate immunocyte populations to produce MMPs (including MMP-9, MMP-13, and MMP-14), which create a favorable environment for HCC progression, a process that is highly conserved [87,88]. Leukocyte-derived MMPs increase tumor invasiveness by remodeling the ECM, while MMP-9 produced by macrophages regulates pathological angiogenesis through VEGF activation [89]. Mechanical stress induces fibroblasts to express pro-inflammatory cytokines such as IL-1α, IL-1β, and IL-6, promoting immune infiltration and linking mechanical stress to inflammation [90]. However, it is not pro-inflammatory macrophages that dominate the myofibroblast-like transition. In pulmonary fibrosis, high expression of integrin αvβ3 by pro-fibrotic macrophages induces intense mechanical stress in fibroblasts, triggering acute cell contraction with the aid of Piezo1 [91]. However, the unresolved “chicken-or-egg” question persists: what is the initiating factor for mechanical stress, immunization, or the metabolic and phenotypic alterations mentioned earlier?

## Mechanosensitive active molecules in the development of CLD

Researchers exposed fibroblasts to conditioned medium from myofibroblasts and found that resting-state fibroblasts did not activate, suggesting that paracrine factors from myofibroblasts alone are insufficient to induce myofibroblast-like transition [91,92]. This suggests that fibroblast activation within liver sinusoids, represented by HSC, is at least partially the result of mechanical-biochemical coupling. In this section, we enumerate a series of classical and newly discovered targets that constitute complex mechanistic signaling networks, which are not inferior to intercellular paracrine signaling in driving mechanotransduction and the strength of biological effects.

### Mechanosensitive ion channel proteins

Mechanosensitive ion channels differ from ligand-gated or voltage-activated channels, as they can be directly activated by mechanical stress [93]. Piezo is a representative mechanosensitive non-selective ion channel responsible for receiving and transducing mechanical and biochemical signals inside and outside cells. In the liver, Piezo1 is widely expressed in fibroblasts, endothelial cells, and immunocytes, and exhibits high sensitivity to lateral membrane tension, with slow decline and inactivation properties [94]. Thus, Piezo1 is involved in HSC activation, endothelial dysfunction, and inflammation in CLD.

Mechanical stress, whether external forces on tissues at the organ level (ECM adhesion) or internal forces generated by intracellular actinomyosin motors [95], can be conveyed as signals of altered membrane tension by caveolae and ion channels [96]. Piezo1 not only directly senses changes in membrane tension triggered by extracellular mechanical stress [68,97], but also amplifies the transmission of intracellular mechanical mechanics signaling triggered by cytoskeleton changes [98]. Multiple mechanistic pathways synergistically drive cation channel (Na^+^, K^+^, Ca^2+^) opening and downstream calpain-2 activation, triggering downstream molecular events [99]. Additionally, ECM cross-linking and tissue stiffness are extremely sensitive to integrin β1 stimulation [72], which activates the membrane-bound Piezo1 via the “intermediary” myosin [100]. Ceramide and polyunsaturated fatty acids produced by sphingomyelin phosphodiesterase 3 (SMPD3) protect Piezo1 from inactivation or reduce the duration of inactivation [101,102].

During the myofibroblast-like transition, Piezo1-activated Ca^2+^ stimulates calmodulin phosphatase and promotes HSC activation by inducing dephosphorylation of NFATc1, YAP1, and β-catenin, forming NFAT/YAP1/β-catenin complexes [103].

LSEC are the fourth largest Piezo1-expressing cell in the liver (relevant data is well summarized on https://bis.zju.edu.cn/HCL/). In particular, Piezo1 at the adherens junction membrane can use membrane tension to drive its own deformation [53], triggering the opening of cation channels and downstream calpain-2 activation [68], after acutely sensing mechanical stress [99]. Accompanied by a massive influx of Ca^2+^, HIF-1α ubiquitination is repressed, and it regulates downstream target gene expression (e.g., Vegf, Igfbp2, Cxcl16) after nuclear translocation, leading to defenestration from a biochemical perspective. Additionally, increased mitochondrial respiration and glycolysis stimulated by Yoda1 (a well-characterized Piezo1 agonist) provide sufficient ATP [104], suggesting that Piezo1 is the “bridge” between mechanical stress and energy metabolism.

**Fig. 7:**
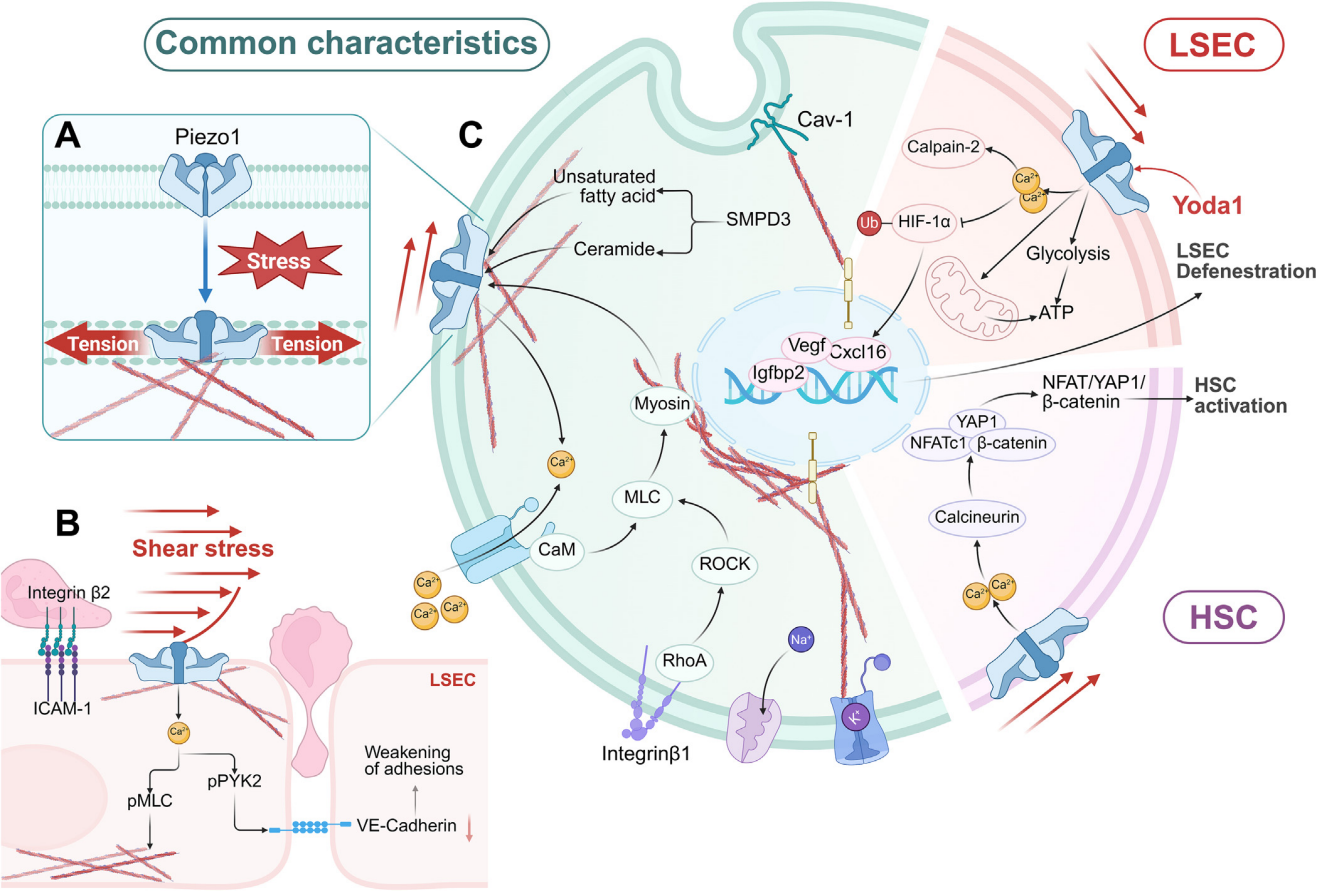
Mechanosensing and transduction functions of Piezo1 in CLD initiation. (A) Piezo1 undergoes conformational changes in response to shear stress and ECM stiffness, transducing mechanical cues and initiating a downstream signaling cascade. (B) Schematic diagram of Piezo1 activation by shear stress, mediating leukocyte extravasation within LSEC. (C) Piezo1′s role in activating major cellular signaling networks in the liver. This figure was Created in BioRender. Tianle, M. (2025) https://BioRender.com/n66c549.

Moreover, Piezo1 also supports inflammation: Aggregation of the leukocyte-induced adhesion molecule ICAM-1 leads to cell surface stiffness, and traction forces generated by the cytoskeleton synergize with blood flow shear to increase plasma membrane tension, activating Piezo1. This triggers downstream phosphorylation of myosin light chain (MLC) and protein tyrosine kinase 2 (PYK2), forming a positive feedback loop with continuous activation of “cytoskeleton-plasma membrane-cytoskeleton mechanosensing” [55], facilitating endothelial barrier opening for leukocyte extravasation. However, some studies have indicated that macrophage cytophagy accelerates the regression of inflammation and fibrosis and is Piezo1-dependent [105], suggesting that immunocytes interactions with Piezo1 may have opposite effects (Fig. 7).

In addition to Piezo1, another channel associated with vascular mechanical and osmotic responses is the transient receptor potential vanilloid 4 (TRPV4) channel. Like Piezo1, TRPV4 is a Ca^2+^-permeable, non-selective cation channel, and is endothelium-dependent, regulating vascular function by responding to shear stress [106]. Unlike Piezo1, TRPV4 is not a simple mechanical sensor but integrates various chemical factors, including arachidonic acid metabolites. Furthermore, TRPV4, located downstream of Piezo1, activates and amplifies intracellular Ca^2+^ signals initially triggered by Piezo1 upon phospholipase A stimulation, leading to NO-dependent relaxation of endothelial cells in the portal vein. However, TRPV4 activation in endothelial cells cannot evoke nitric oxide synthase 3 (NOS3) or SK/IK Ca^2+^-dependent relaxation mechanisms. When intense mechanical stress and shear stress occur, the effect of TRPV4 is suppressed by Piezo1, which predominates [50].

### Integrin and FAK

The mechanism by which fibroblasts respond to ECM stiffness is divided into two steps: mechanosensing and mechanotransduction. Mechanosensing is primarily governed by integrins, while mechanotransduction is regulated by focal adhesion kinase (FAK), which synergistically combines mechanical stress alteration with biochemical signaling pathway activation [107]. Integrins are transmembrane proteins that dimerize, consisting of α and β subunits, and are expressed in all liver cells [108]. Integrins serve as a “transportation hub” that transduces mechanical stress between the inside and outside of cells, in addition to Piezo1, which senses shear stress, ECM stiffness, and cytoskeletal traction, thus participating in HSC myofibroblast-like transitions [109] and vascular anomalies [17]. FAK, a protein tyrosine kinase, mediates cell adhesion to the ECM [110] and directs the migration of fibroblasts toward areas of higher stiffness [111]. Together, they form an “ECM-integrin-FAK-actin” mechanotransduction system, transmitting mechanical signals from the outside to the inside. The cross-linking stiffness of ECM and the mechanical stress generated by actin’s autonomous contraction trigger an “activation process” in integrins [112] (transitioning from a low-affinity bent closed state to a high-affinity extended-open state) [113], altering the activity and conformation of FAK [39], and allowing mechanical signals to be transmitted to the cytoskeleton [114].

**Fig. 8:**
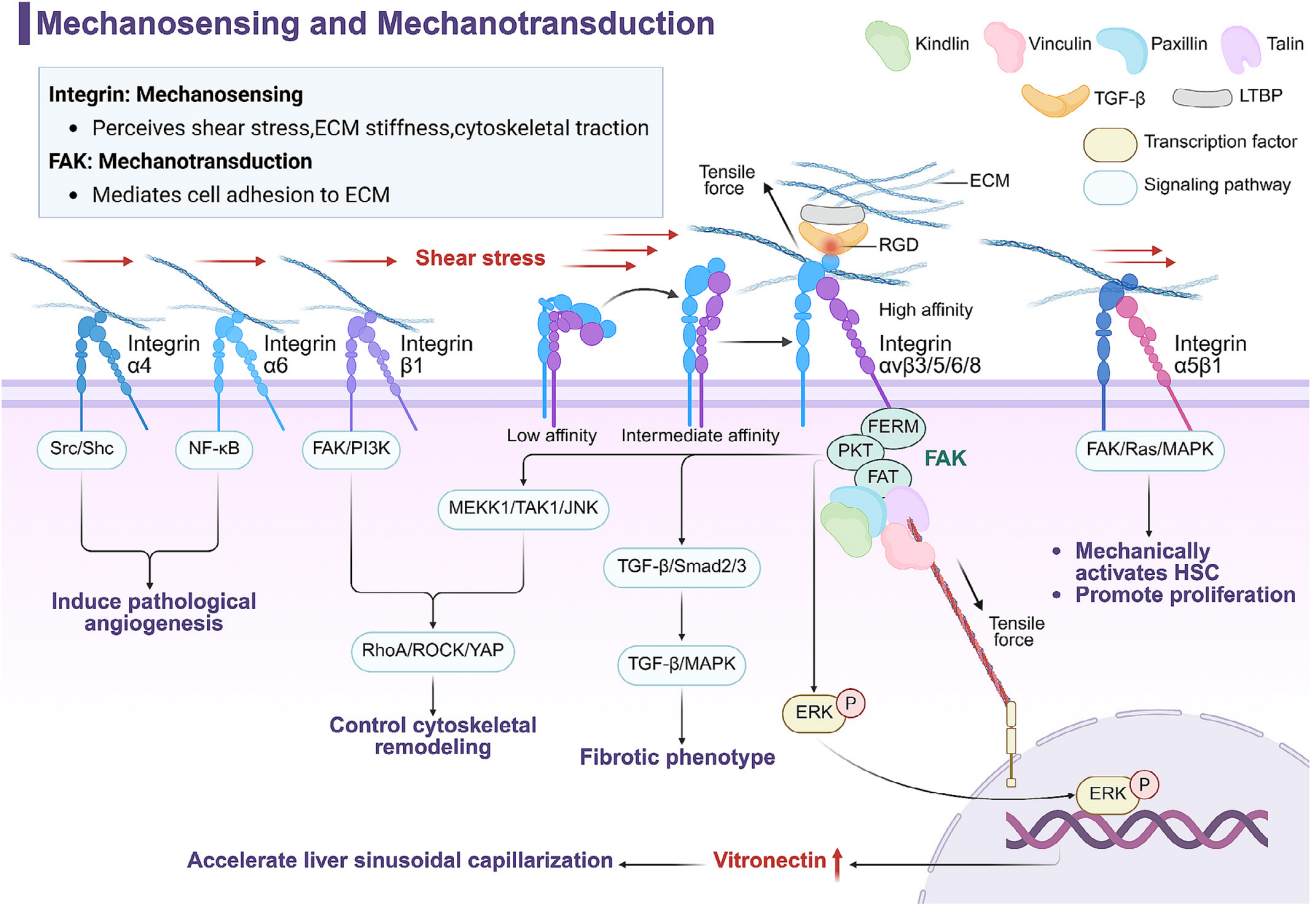
The role of different integrin subunits in mechanosensing, influencing cellular phenotype and physiological functions. This figure was created in BioRender. Tianle, M. (2025) https://BioRender.com/i46f555.

Different subunits of integrin exhibit various pathogenic characteristics during CLD. For example, integrin α4 and α6 induce pathological angiogenesis through Src/Shc and NF-κB signaling pathways after shear stress activation [17]. Integrin β1 also regulates cytoskeletal remodeling through the FAK/PI3K pathway and downstream RhoA/ROCK and mDia pathways [115]. Often, integrin subunits act in a dimerized form; for example, αv [116] heterodimerizes with β3, β5, β6, or β8 [117] subunits that bind to RGD sequences in the structural domain of TGF-β1 [118]. This converts mechanical stress into biochemical signals that activate the TGF-β/Smad2/3 and TGF-β/MAPK signaling pathways, promoting a fibrotic phenotype. In addition, non-αv subunits such as α5β1 also aggregates upon sensing ECM stiffness, mediates adhesion complex formation, mechanically activate HSC via FAK/Ras/MAPK, and promote proliferation [119] (Fig. 8).

Integrin-activated FAK promotes pro-fibrotic gene expression via the MEKK1/TAK1/JNK-driven pathway in myofibroblasts [120], in conjunction with the downstream RhoA/ROCK/YAP pathway [27]. FAK interacts with death receptors and effectively avoids apoptosis in myofibroblast-like cells through p53 degradation [39]. Integrin αvβ5/FAK also enhances nuclear translocation of phosphorylated ERK, increases expression of vitronectin, a basement membrane component of LSEC, and accelerates liver sinusoidal capillarization [121].

If Piezo1 is the “receptor” of mechanical stress, then integrin/FAK is the “afferent nerve” of intracellular mechanotransduction that mediates the “pain” response of cells in liver sinusoids. Targeting integrin/FAK can significantly alleviate liver damage from mechanical stress (some prospective strategies are listed in Section 4.1).

### β-arrestin2

Arrestins inhibit signaling by G protein-coupled receptors (GPCRs). Among the four family members, only arrestin2 and arrestin3 are expressed in the liver, and β-arrestin2 is positively associated with fibrosis progression. In the Ang II/AT1R/ROCK mechanical mechanics conductance axis, β-arrestin2 displays a significantly higher affinity for β2-adrenergic receptors than its companions, suggesting that β-arrestin2 may profoundly influence the progression of CLD [[122], [123], [124]].

CXCR4 is a GPCRs with prominent roles in liver cirrhosis progression and HCC metastasis [125]. It facilitates proliferation and migration through the RhoA/ROCK/p38 MAPK pathway, cellular chemotaxis though the PI3K/ Paxillin/FAK pathway, and upregulates gene expression related to cell proliferation and angiogenesis through the Ras/ERK1/2 pathway. For instance, GPCR kinases such as GRK6 and GRK3 can modify the β-arrestin2 conformation through CXCR4 phosphorylation, enabling full activation of ERK1/2 [123]. In contrast, β-arrestin2 directs CXCR4 to the lysosome for degradation, thus inhibiting mechanotransduction downstream of GPCR. Moreover, β-arrestin2 sequesters calmodulin, preventing it from remodeling the cytoskeleton by binding to microtubules, though this action is Ca^2+^-dependent [123]. Notably, in patients with PH and ascites treated with nonselective β-receptor blockers, β-arrestin2, characterized as a negative regulator of the RhoA/RocK2 pathway downstream of the RAS system (AT1R), may serve as a valuable biomarker for detecting hemodynamic changes [124,126].

Angiotensin II type 1 receptor (AT1R) and endothelin-1 type A receptor (ET_A_R) are members of the GPCR family [127]. Both receptors can inhibit mechanotransduction downstream of Ang II/AT1R by binding to β-arrestin2 and undergoing cytosolization [128]. In contrast, β-arrestin2 positively regulates NOX4 expression, exacerbating Ang II-stimulated ROS and α-SMA expression in HSC, which is attributed to β-arrestin2′s promotion of the Ras/ERK1/2 and JNK signaling pathways [128,129]. Thus, β-arrestin2 knockdown enhances type III TGF-β receptor expression and inhibits TGF-β1/Smad signaling, attenuating the mechanical stress associated with ECM deposition [130]. β-arrestin2 may also influence ECM synthesis in HSC by regulating the PI3K/AKT pathway. Notably, the Wnt/β-catenin pathway promotes collagen contraction, α-SMA expression, and cell migration in human dermal fibroblasts [124]. However, it remains unclear whether β-arrestin2 serves as a positive regulator in CLD.

The effects of β-arrestin2 on other cells primarily involve GPCR downstream molecules, such as enhancing focal migration and macrophage infiltration through activation of the Ras/ERK1/2 and p38 MAPK signaling pathway, and promoting the differentiation of monocyte THP-1 into macrophages, which exacerbates the immune burden of the liver [131].

Based on the above studies, in the β-arrestin2/GPCR network, β-arrestin2 acts as the central node [132], with numerous GPCRs participating in cellular functions such as gene transcription, pro-inflammatory responses, and cytoskeletal remodelling. However, regulating β-arrestin2 expression to maintain the functional level required for normal survival without disrupting mechanical homeostasis and biochemical equilibrium poses a challenging issue that has not yet been fully resolved.

### TGF-β1

TGF-β is expressed in various liver cells, and among its three isoforms, TGF-β1 is the strongest pro-fibrotic cytokine [133]. Newly secreted TGF-β1 does not act immediately; instead, it binds to the latency-associated peptide (LAP) to form LAP-TGF-β1 dimers [134], which are stored in the ECM “reservoir” until integrins sense ECM stiffness and deliver traction to disrupt the LAP conformation (>40 pN traction [45]), or until plasma kallikrein cleaves LAP at arginine and lysine residues [135], allowing TGF-β1 to be released and activated [39].

HSC is the primary site of TGF-β1 pro-fibrosis, and TGF-β1 promotes myofibroblast-like transition through the Smad2/3 pathway [27]. It also regulates proliferation, migration, differentiation, and apoptosis via non-Smad-dependent signaling pathways, including MAPK, mTOR, PI3K/AKT, and Rho-GTPase/ROCK [27,136]. In LSEC, TGF-β1 induces endothelial-to-mesenchymal transition (EndMT), leading to cirrhotic decompensation. Bone morphogenetic protein-7 (BMP-7), also a member of the TGF-β family, inhibits fibrosis and pathological angiogenesis by suppressing EndMT [137]. Additionally, negative regulators Smad6 and Smad7 inhibit the TGF-β1 signaling axis by competing for TβRI [138] and have shown potential efficacy in cardiac and renal fibrosis [137]. TGF-β1-activated LSEC also exhibits inhibitory effects on BMP2 and BMP6, leading to senescence and apoptosis via paracrine secretion with HSC and hepatocyte iron overload [138]. Despite current findings, strategically manipulating the expression and functional balance between TGF-β family members to suppress CLD progression remains a significant challenge.

The pro-cancer effects of TGF-β1 after cirrhotic decompensation can be summarized as follows: (1) Production of various tumor-promoting factors through activation of FAK, including programmed cell death 1 ligand 1 (PD-L1), insulin growth factor-1 (IGF-1), and fibroblast growth factor-2 (FGF-2). Notably, IGF-1, in combination with its receptor, mediates Nanog protein expression, directly promoting the formation of cancer stem cells in HCC [139]. (2) Formation of an inhibitory immune microenvironment: TGF-β1 can suppress T cell and NK cell activity [140]. (3) Promotion of tumor cell invasion: TGF-β1 stimulates PDGF and CTGF production along with MMPs to degrade the ECM [141]. (4) Promotion of tumor cell EMT leading to migration, extravasation, and metastatic spreading [142]. These phenomena fully confirm the synergistic cancer-promoting effects of mechanical stress and biochemical signaling.

### Ca^2+^

Ca^2+^ serves as an intracellular second messenger, and its cytoplasmic level is influenced by concentration gradients inside and outside cells, the opening of calcium channels, and uptake/release from the endoplasmic reticulum (ER), the intracellular “calcium reservoir” [143]. The release of Ca^2+^ is stringently regulated, with IP3R and ryanodine receptor mediating Ca^2+^ release from the ER/SR. Ca^2+^ channels and transport proteins, such as voltage-gated Ca^2+^ channels, Na^+^/Ca^2+^ exchangers, Piezo1, and TRPV4, regulate Ca^2+^ influx [144]. Specifically, mechanosensitive ion channels like Piezo1 and TRPV4 can detect membrane tension, opening within one second and stabilizing after 5 s to permit Ca^2+^ influx [92]. These dynamics are involved in cellular processes including proliferation, migration, and nearly all physiological and pathological processes in the liver.

Upon hypertonic stimulation within the fibrotic environment, Ca^2+^ forms a complex with calmodulin (CaM) and interacts with CaMKII. The γ subunit of CaMKII, which is highly expressed in the liver [145], exhibits both positive and negative regulatory effects on fibrosis. In activated HSC, Ca^2+^ concentration notably increases in response to vasoactive molecules or mechanical stress [27]. The TGF-β1/Smad pathway is one of the downstream signals regulated by Ca^2+^, where TGF-β1 activation leads to nuclear translocation of the Smad2/3/4 complex, overexpressing MMP-2, MMP-9, and MMP-14, thus remodeling the ECM [141]. Another mechanical clue that mediates the fibrotic phenotype in HSC involves Ca^2+^/CaM/MLC-induced cytoskeletal remodeling with YAP nuclear translocation [27]. Non-Smad-dependent TGF-β1/MAPK pathways also increase after Ca^2+^ stimulation, elevating ER stress markers GRP-78 and caspase-12 [136], while clearing activated HSC through stress or ferritinophagy has proven effective in treating fibrosis [9]. Differently, Ca^2+^-activated PDGF/ERK1/2 downregulates cell cycle inhibitory regulators p53 and p21, thereby promoting HSC proliferation [146].

Clinically, Ca^2+^ channel blockers such as Nifedipine and Verapamil are commonly used as adjunctive therapy for PH and demonstrate improved prognosis for CLD [147,148]. However, Ca^2+^ channel blocking drugs cannot specifically target organs or cells, nor can they interfere with intracellular ER/SR Ca^2+^ release, often a factor in cell apoptosis due to Ca^2+^ overload inducing ER and mitochondrial stress [149]. The concept of using nanomedicine for targeted co-delivery of Ca^2+^ channel blockers may provide reference [150], but precise regulation of intracellular Ca^2+^ activity remains elusive and demands further experimental elucidation.

### YAP/TAZ

**Fig. 9:**
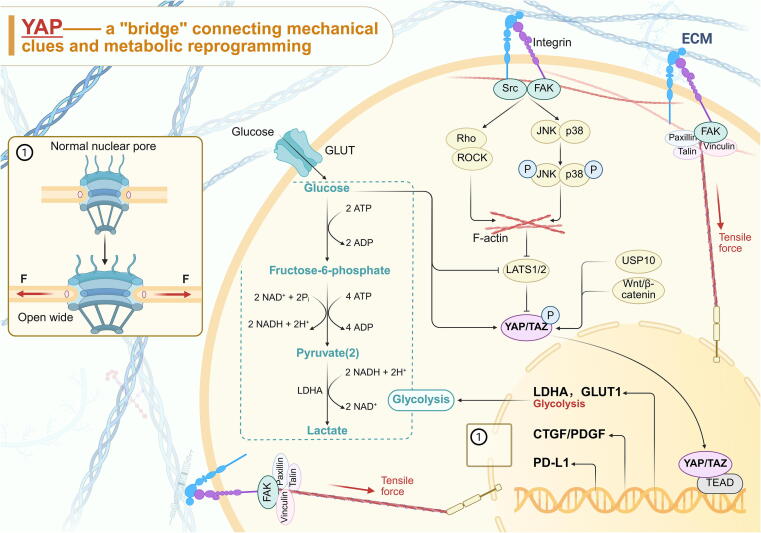
The influence of mechanically activated cytoskeletal remodeling: YAP regulates various transcriptional programs upon nuclear translocation and serves as a key effector of mechanical cues bridging the nucleus. This figure was created in BioRender. Tianle, M. (2025) https://BioRender.com/f88a751.

Yes-associated protein (YAP)/TAZ is a downstream effector of the Hippo pathway, activated by integrin-sensing mechanical cues (including ECM stiffness, cell geometry, and cell–cell contacts) [151] coupled with biochemical signals (including TGF-β, Wnt/β-catenin, MAPK/ERK, and NF-κB) [26]. Inhibition of LATS1/2-mediated phosphorylation at YAP Ser127 and 397 sites enables YAP nuclear translocation [152]. Interestingly, aside from biochemical factors, the cytoskeleton, stimulated by mechanical cues (physical factors), can directly pull on nuclear pores, prompting them to open wide and allowing dephosphorylated YAP to pass through [27]. In the nucleus, YAP interacts with TEAD family members to facilitate the promotion of target gene expression. This phenomenon positions YAP as a “bridge” between mechanical cues and metabolic reprogramming, dependent on two pillars: RhoA/ROCK and cytoskeletal integrity [151], which in turn affects liver homeostasis, regeneration, and even HCC occurrence and metastasis. Sufficient evidence indicates that YAP nuclear translocation in HSC, synchronized with myofibroblast-like transition, controls proliferation and ECM synthesis via CTGF and PDGF, becoming a hallmark event in CLD [27,153,154]. Conversely, during liver injury from ischemia-reperfusion, intranuclear enrichment of YAP predicts favorable regenerative repair. Paradoxically, HSC activation itself is a necessary condition for systemic injury repair. In the short term, YAP activation appears to drive beneficial non-cell autonomous effects, but in the long term, it exacerbates fibrosis and cirrhosis [155]. YAP/TAZ also serves as a mechanical cue for VEGF expression and is necessary to maintain a normalized liver sinusoidal vascular network [152]. Mechanical stress-induced YAP nuclear translocation prompts hepatocyte dedifferentiation and increases LDHA and GLUT1 expression, promoting aerobic glycolysis in hepatocyte during the HCC period [156]. Simultaneously, YAP/TAZ also facilitates the exchange of the hepatocyte glutamine metabolite glutamate with cancer-associated fibroblasts (CAFs)-derived aspartic acid, fulfilling the expansion needs of hepatocyte and maintaining the function of CAFs in remodeling ECMs [157]. Given that glycolysis directly sustains the transcriptional activity of YAP/TAZ, a positive feedback loop is formed (Fig. 9). Overall, therefore, inhibiting YAP in the terminal phase is beneficial for salvaging liver function, both mechanistically and through metabolic crosstalk [151,158].

In addition to the widely reported mechano-metabolic crosstalk, YAP also promotes cancer cell proliferation through the deubiquitinating enzyme USP10 and Wnt/β-catenin activation [159]. Moreover, YAP regulates PD-L1 to foster tumor immune escape and drug resistance [160]. Given YAP/TAZ's multifaceted roles, this pathway presents multiple promising targets for clinical development [27,151].

### MRTF

Myocardin-related transcription factor (MRTF) is a transcriptional co-activator [161] and a mechanical mediator of myofibroblast-like transition. In a normal liver, MRTF binds to G-actin and remains in the cytoplasm. However, when ECM conveys mechanical stress to RhoA/ROCK via integrin, promoting cytoskeletal remodelling, or when TGF-β induces the polymerization of G-actin to F-actin [162,163], MRTF releases G-actin and undergoes nuclear translocation. This drives the transcription of additional cytoskeletal and ECM components, such as Acta1, Actg2, and Col1a1, resulting in the assembly of denser stress fibers [[163], [164], [165]], marking activated HSC [27]. Interestingly, MRTF may exhibit some dependency or competitive interaction with YAP effector TAZ and Smad3 downstream of TGF-β in the nucleus [166]. In HCC cells, ECM remodeling enhances MRTF conductance, which, coupled with activation of the ECM/RhoA/ROCK/YAP signaling pathway, significantly promotes proliferation, migration, and dissemination [167,168], forming disseminated cancer cells [167].

## New drug development strategy for anti-CLD based on mechanical mechanics conduction in cellular perspective

**Fig. 10:**
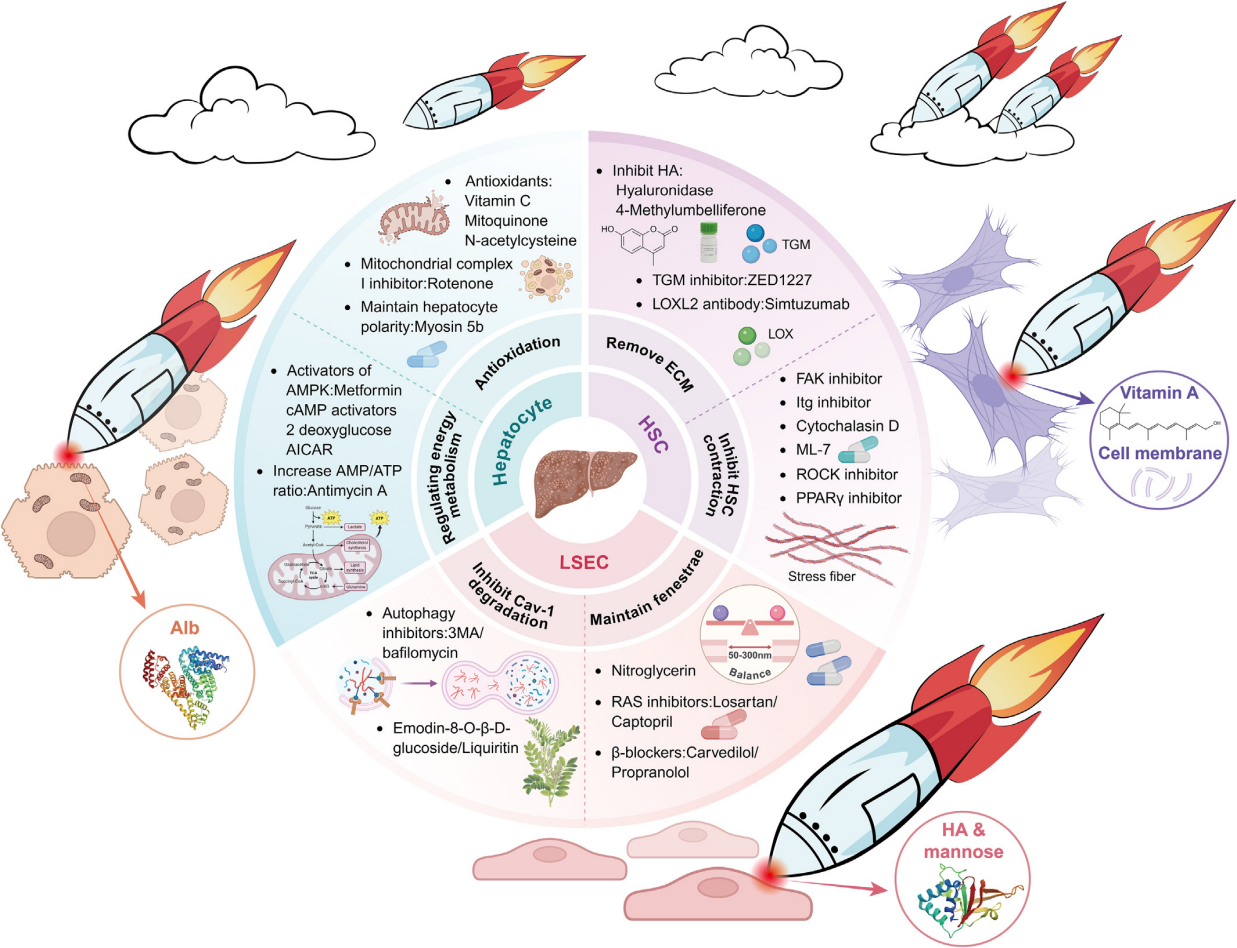
Potential mechanical-biochemical coupling targets and therapeutic strategies for CLD from the perspective of HSC-hepatocyte-LSEC. A series of modification or encapsulation methods, like the warhead in the front of the missile, can achieve precise targeted removal of target cells. This figure was created in BioRender (Tianle, M. (2025) https://BioRender.com/9ppi7p3) and Adobe Illustrator 2024.

In the context of fibrosis, both hepatocytes and nonparenchymal cells experience varying degrees of mechanical stress, leading to hepatocyte necrosis, HSC activation, and LSEC dysfunction. As fibrosis progresses to the end stage, nonphysiological mechanical stress (manifested as portal hypertension in the liver) can lead to ascites and the formation of portal vein anastomotic branches, indicating a vicious cycle of decompensated hemodynamic disorders and potential cancer. Unfortunately, few drugs or means exist to treat abnormalities in the liver sinusoidal microenvironment during the compensated phase of CLD. Due to the liver's robust reserves, patients often lack sufficient awareness and subjective perception of their condition, coupled with poor compliance to long-term medication use, thus overlooking the continued progression of CLD until decompensation occurs. Knowledge about the key regulatory links affecting the development of CLD is not lacking. From the perspective of mechanical-biochemical coupling, targeting direct or indirect links of mechanoregulation to achieve dual-targeted therapy is both feasible and promising (Fig. 10). Clinical trials of related drugs are just a step away from structural modification, delivery design, and other critical steps.

### HSC

We have previously summarized therapeutic strategies targeting biochemical factors in HSC. After systematically elucidating the mechanical cues of HSC myofibroblast-like transition, we will also divide the strategies targeting HSC mechanics into two aspects.

First, the most apparent characteristic of myofibroblast-like transition is the fibroblastic phenotype caused by ECM deposition. Targeted removal of deposited ECM is the most direct and unavoidable strategy. HA, LOX, TGM, and MMPs are the main contributors to ECM cross-linking, stiffness, and deposition, and ECM deposition not only exerts mechanical stress on the ecological niche of liver sinusoidal cells, but also serves as an “umbrella” for cells to evade drug delivery therapy. ECM degradation is therefore a versatile and effective option for improving fibrotic diseases, sclerosis, and even cancer (Table 2). The most straightforward approach to targeting HA is to inhibit its production. 4-Methylumbelliferone depletes HA precursors [169], although it lacks clinical trial validation. Another approach is to fragment HA through a PEGylated recombinant human hyaluronidase or by utilizing envelope delivery of hyaluronidase [90,170]. However, this may pose a potential risk of angiogenesis and disruption of vascular integrity [171]. Simtuzumab, a targeted LOXL2 humanized monoclonal antibody, failed to demonstrate efficacy in liver fibrosis. The TGM inhibitor ZED1227 is also in phase II trials. The delivery system that loads NO to induce MMP activation and degrade ECM represents a groundbreaking idea [172].

**Table 2:** Promising drugs for removing ECM deposition.

No.	Drug name	NCT Number	Target	Indication	Current status
1	4-methylumbelliferone	00225537	hyaluronic acid synthase	Chronic Hepatitis B; Chronic Hepatitis C	Phase Ⅱ
2	Limbrel	00928837	COX/5-LOX	Osteoarthritis	N/A
3	Thymol	05427721	COX-2/5-LOX	Inflammatory Response; Obesity	Phase Ⅱ
4	Zileuton	01805687	5-LOX	Asthma	Phase Ⅳ
5	MiRNA-30a	03235128	LOX	Nephrotic Syndrome Steroid-Resistant	Observational
6	Curcumin	05753436	LOX-1	Diabetes Mellitus, Type 2; Dyslipidemias; Hypertension	Phase Ⅱ
7	ZED1227	05305599	TG2	NAFLD; Liver Fibrosis	Phase Ⅱ
8	Mercaptamine	04246060	TG2	Nephropathic Cystinosis	Observational
9	Marimastat	00002911	MMP-1/-2/-3/-7/-9	Lung Cancer	Phase Ⅲ
10	BMS-275291	00024024	MMP-9	Sarcoma	Phase Ⅰ/Ⅱ
11	Prinomastat	00003343	MMP-2/-3/-9/-13	Prostate Cancer	Phase Ⅲ
12	Minocycline	06307288	MMP-9	Rosacea	Phase Ⅳ
13	Bortezomib	06505369	MMP-2/MMP-9	Multiple Myeloma	Phase Ⅱ
14	HYADD	02187549	Hyal-2/MMP-13	Knee Osteoarthritis	N/A

Secondly, abnormal contraction of HSC caused by cytoskeleton remodeling is addressed by intervening upstream of the mechanical cues. Inhibitors targeting integrin and FAK have been in development for treating various fibrotic and cardiovascular diseases for over 20 years (Table 3). The cutting-edge and most promising ones, such as the FAK inhibitor Defactinib, have shown better antifibrotic effects in pancreatic cancer patients in phase II trials [39], although evidence for CLD is still lacking. Targeting ROCK downstream of integrin has demonstrated efficacy, but ROCK inhibitors require more precise organ or tissue cell-targeted delivery due to the potential risk of systemic hypotension [39]. Furthermore, utilizing Cytochalasin D and the MLC kinase inhibitor ML-7, which block the polymerization of G-actin and the assembly of stress fibers, respectively, could effectively intervene in cytoskeletal remodeling at the ends of mechanical cues [27], although these drugs have yet to pass rigorous clinical trials. From the case of nerve injury [75], we speculate that targeting PPARγ to inhibit lipid oxidation also inhibits downstream RhoA/ROCK/cytoskeletal remodeling, a strategy applicable to HSC (opposite regulatory strategy may apply to hepatocyte). If the restoration of lipid droplet synthesis can be accompanied by the reduction of GLUT1 transport of glucose and the inhibition of lactate accumulation caused by glycolysis, a dual reprogramming of metabolism by mechanical-biochemical multi-pathways may be achieved. If the targeted treatment of HSC is compared to the targeted removal of missiles, we have completed the loading of the missile's “warhead.” Next, GPS positioning needs to be installed for the missile. The mainstream approach involves modifying the delivery system with vitamin A or using the HSC cell membrane to coat nanomedicines. This is due to the high expression of retinoic acid receptors and retinol-binding protein receptors on the HSC surface [173]. Vitamin A can specifically bind to HSC through these receptors, achieving active targeting via endocytosis [174]. Cell membrane modification can also enhance homologous targeting significantly [175]. However, this may be more effective for quiescent HSC, as vitamin A is consumed during the myofibroblast-like transition, which largely affects the accuracy of the delivery system. The missile could miss its target and fall into a “civilian area” if not carefully managed.

**Table 3:** Targeted agents against integrin or FAK.

No.	Drug name	NCT Number	Target	Indication	Current status
1	Natalizumab biosimilar	04115488	α4β1; α4β7	Relapsing-remitting multiple sclerosis	Phase Ⅲ
2	Abrilumab	01694485	α4β7	Ulcerative colitis	Phase II
		01696396	α4β7	Crohn's Disease	Phase II
3	PN-943	04504383	α4β7	Ulcerative colitis	Phase II
4	PTG-100	02895100	α4β7	Ulcerative Colitis	Phase II
5	THR-687	05063734	α5β1	Diabetes Mellitus; Diabetic Macular Edema	Phase II
6	ASP-5094	03257852	α9β1	Rheumatoid Arthritis	Phase II
7	Intetumumab	00246012	pan-αv	Melanoma	Phase II
8	Abituzumab	01008475	pan-αv	Metastatic Colorectal Cancer	Phase I/II
9	BG-00011	03573505	αvβ1; αvβ6	Idiopathic Pulmonary Fibrosis	Phase II
10	PLN-74809	06097260	αvβ1; αvβ6	Idiopathic Pulmonary Fibrosis	Phase II
11	AXT-107	04746963	αvβ3; α5β1	Neovascular Age-Related Macular Degeneration	Phase I/II
12	18F-FPPRGD2	02995642	αvβ3	vascular inflammation	Phase II
13	CEND-1	05042128	αvβ5	Metastatic Pancreatic Cancer	Phase II
14	E-7820	05024994	α2	Acute Myeloid Leukemia	Phase II
15	ATL-1102	05938023	α4	Duchenne Muscular Dystrophy	Phase II
16	VVN-001	04556838	LFA-1A	Dry eye disease	Phase II
17	7HP-349	04508179	αLβ2; α4β1	Solid Tumor	Phase Ⅰ
18	OS2966	04608812	β1	Glioma	Phase Ⅰ
19	SGN-B6A	04389632	β6	Solid tumors	Phase Ⅰ
20	CAR- T therapy	03778346	β7	Relapsed/refractory multiple myeloma	Phase Ⅰ
21	Tirofiban (AGGRASTAT)	06587347	αⅡbβ3	Acute Stroke; Cerebral Infarction	Phase Ⅰ/Ⅲ
22	Zalunfiban	04825743	αⅡbβ3	ST-elevation Myocardial Infarction	Phase Ⅲ
23	PF-04554878	00787033	Pyk2	Cancer	Phase Ⅰ
24	Conteltinib (CT-707)	02695550	pan-FAK	Non-small Cell Lung Cancer	Phase Ⅰ
25	GSK-2256098	01138033	Tyr397	Cancer	Phase Ⅰ
26	CEP-37440	01922752	FAK/ALK	Solid Tumors	Phase Ⅰ
27	PF-00562271	00666926	FAK/Pyk2	Head and Neck Neoplasm; Prostatic Neoplasm; Pancreatic Neoplasm	Phase Ⅰ
28	APG-2449	03917043	ALK/ROS1/FAK	Advanced Solid Cancer; Non Small Cell Lung Cancer	Phase Ⅰ
29	GSK2256098	01938443	Tyr 397	Cancer; Neoplasms	Phase Ⅰ
30	BI 853520	01335269	FAK	Neoplasms	Phase Ⅰ

### Hepatocyte

From the “chicken or egg” question in “2.3.3”, we recognize that although there is no shortage of drugs to treat liver injury from various etiologies [176,177], patients in clinical practice often have multiple underlying conditions. Taking a variety of drugs can theoretically treat the disease, but it remains a significant challenge for patient compliance due to potential drug interactions. Therefore, finding common links across different etiologies at the cellular level is a key question worth considering.

In terms of mechanical mechanics pathogenic features, both ECM stiffness in the interstitial space and poor circulation due to intrahepatic vascular resistance (IHVR) contribute to hepatocyte ischemia and hypoxia. This, in turn, triggers a cascade of reactions including a sudden increase in ROS expression, stimulation of mitochondrial autophagy, an inflammatory storm, and hepatocyte apoptosis [178]. In this context, some antioxidants such as Vitamin C [179], Mitoquinone [180], N-acetylcysteine, and the mitochondrial complex I inhibitor, Rotenone [178], demonstrate complete inhibition of human hepatocyte autophagy. The conversion and maintenance of hepatocyte polarity rely on the mechanotransduction of ECM, cytoskeleton, and energy supply. Myosin 5b, a classical cytoskeletal molecular motor encoded by the gene RE, maintains hepatocyte polarity. Mutations in myosin 5b are often associated with transporter protein defects in hepatocyte polarity disorders [79]. However, of the 30 different myosin 5b identified, those that reduce intestinal bile acid absorption capacity may improve CLD [181]. Since normal expression of myosin 5b is CaM-dependent, and 10 μM Ca^2+^ significantly increases dissociated CaM [182], it is feasible to control intracellular Ca^2+^ concentration or inhibit CaM dissociation.

Hepatocyte polarization is necessarily energy-dependent due to various mechanical-biochemical couplings. AMPK acts as a mechanical-metabolic sensor [183] to adjust energy supply and expenditure by sensing the cellular AMP/ATP ratio. Metformin, cAMP activators, 2 deoxyglucose, AICAR, or taurocholate are all potent activators of AMPK [79]. Rotenone and Antimycin A can induce AMP accumulation, increase the AMP/ATP ratio to activate AMPK, regulate mitochondrial dynamics (fusion and fission), and ensure sufficient ATP production [183]. Overall, mechanical mechanics interventions can affect inflammation, energy metabolism, and other components centered on the cytoskeleton to maintain hepatocyte homeostasis. Albumin is highly expressed in hepatocytes and is also a common promoter sequence used in liver-targeted gene intervention technologies [184]. It is feasible and accurate to modify albumin on the surface of the delivery system [185].

### LSEC

Currently, it is understood that LSEC defenestration is primarily associated with Cav-1 degradation and F-actin remodeling. Researchers have made a series of targeted attempts. Autophagy inhibitors such as 3MA and bafilomycin can reduce Cav-1 degradation and even reverse defenestration [59]. In addition, maintenance of fenestrae is dependent on the eNOS/NO pathway. As vascular endothelial cell, LSEC is highly receptive to NO. Emodin-8-O-*β*-D-glucoside and Liquiritin, both monomer components isolated from natural plants, target the MK/integrin/α6/Src signaling pathway, increase NO production, and dilate liver sinusoidal blood vessels while maintaining fenestrae [17,19]. Exogenous NO supplementation therapies, such as commonly used oral Nitroglycerin [186], intravenous sodium nitroprusside [187], or novel NO delivery systems and specific release [188], provide significant improvement in liver injury and other ischemic disorders, and may offer insights for NO restoration of fenestrae. Although VEGF within LSEC is beneficial for maintaining fenestrae and the cytoskeleton, in the context of the liver, it can lead to abnormal tumor vessels and promote carcinogenesis. Clinical first-line agents such as Sorafenib [19] and Bevacizumab [189] target VEGF but are clearly at odds with therapy for LSEC. Balancing VEGF expression in different stages of CLD and HCC remains an unanswered challenge. RAS inhibitors such as Captopril [190] and Losartan [191] can inhibit downstream effector aldosterone production, alleviate autophagy, and restore fenestrae [60]. Carvedilol and Propranolol, FDA-approved β-blockers, are effective in ameliorating various causative factors that lead to LSEC defenestration. Particularly, Carvedilol is not only used in the treatment of PH [192] but also offers benefits such as scavenging oxygen free radicals and protecting mitochondrial function [193]. In the case of reducing toxicity and increasing efficiency, LSEC, with high expression of HA [194] or mannose receptors [195] can be considered for design. However, LSEC is a special endothelial cell, making it difficult to completely distinguish it from vascular endothelium.

## Conclusion and prospect

The narrative begins with three representative mechanical cues, tracing key links and small molecules to elucidate the micromechanical factors of liver dysfunction, and culminates in rescue analysis using single cells as a model. The contribution of mechanical stress to the adverse outcomes in liver homeostasis disruption is undisputed, regardless of the underlying cause. We have effectively highlighted the developmental patterns and potential pathways of mechanical-biochemical coupling in the pathogenesis of CLD and HCC. However, compared to clinical biochemical markers, direct or indirect indicators of mechanical stress remain scarce, and direct treatment strategies for the metabolic and immune crosstalk driven by mechanical stress are still lacking. Furthermore, several questions remain: Does targeting fibroblasts and ECM ecological niches influence primary tumor maturation, growth, metastatic escape, and dissemination? How can we precisely regulate the expression of specific proteins within different cells to meet functional needs without disrupting the mechanical-biochemical balance? The development of multicellular synergistic therapies, mechanical-biochemical coupling, and precision-targeted delivery platforms represent effective strategies for treating fibrotic and sclerotic diseases, opening new avenues for managing chronic complex conditions.

## Compliance with Ethics Requirements

*There were no experimental animal or human studies.*

## Declaration of competing interest

*The authors declare that they have no known competing financial interests or personal relationships that could have appeared to influence the work reported in this paper.*
